# Congruence of Effective Leadership Values between Nurse Leaders and Staff Nurses in a Multicultural Medical City in Saudi Arabia: A Sequential Mixed-Methods Study

**DOI:** 10.3390/healthcare11030342

**Published:** 2023-01-25

**Authors:** Ian Flor Flores, Wireen Leila T. Dator, Jennifer Joy Olivar, Mastoura Khames Gaballah

**Affiliations:** 1 College of Nursing and Allied Health Sciences, St. Paul University Manila, 680 Pedro Gil St. Malate, Metro Manila 1004, Philippines; 2 Department of Medical-Surgical Nursing, College of Nursing, Princess Nourah Bint Abdulrahman University, Riyadh 11671, Saudi Arabia; 3 Almoossa College of Health Science, Alhasa Almubarize 8021, Saudi Arabia

**Keywords:** value congruence, nurse leaders, staff nurses, multicultural, diversity, patient safety

## Abstract

This study explores the perceived congruence of effective values of nursing leadership between the nurse leaders and the staff nurses in a multicultural tertiary hospital. Methods: This is a descriptive sequential mixed-methods study conducted in a multicultural medical city in Saudi Arabia. Purposive sampling was used in the qualitative phase, while stratified sampling was used for the quantitative part. There were 70 participants in the qualitative phase, including 33 nurse leaders and 37 staff nurses. The quantitative phase had 571 participants, including 105 nurse leaders and 466 staff nurses. Results: Congruent values were categorised into six emerging themes: (1) cascading deference; (2) paragon of probity; (3) professional competence; (4) compassionate presence; (5) team diversity and inclusion; (6) calibrated communication. The quantitative survey confirmed that the values identified from the interviews were considered to be vital by both nurse leaders and staff nurses, and there were no statistically significant differences between staff nurses’ and nurse leaders’ perceptions, regardless of their nationality, as demonstrated by one-way ANOVA, with *p*-values less than 0.05 deemed to be statistically significant. Conclusion: Both nurse leaders and staff nurses in the multicultural institution have congruent leadership values that are perceived as essential to achieve institutional goals and, ultimately, safe and high-quality patient care.

## 1. Introduction

Healthcare, including nursing practice, is heavily value-laden and relies greatly on leader–subordinate teamwork to ensure high-quality and safe patient care [1,2,3]. This becomes much more complicated in a multicultural milieu, as found in most tertiary hospitals in Saudi Arabia, where the medical and nursing human resources are a mix of several nationalities with varied cultures, languages, and personal and professional values. Differences in language, culture, and even values sometimes hinder the smooth, effective, and efficient delivery of services and daily operations in an institution. It is imperative to ensure the presence of a common zone where leaders and staff can meet and collaborate regardless of their differences. Balance in the beliefs between followers and representatives can be achieved through value alignment [4]. Leaders who can inspire their followers to pursue their goals and aspirations and persuade them to strive to achieve their organisational objectives are more effective at doing so [5]. Leaders might be more certain that their followers will carry out the organisation’s goals, objectives, and dreams if they hold similar values [6]. Shared values between leaders and followers can motivate the followers to achieve organisational success [7].

Schwartz (2004) [8] concluded that organisations’ inability to establish congruence between leader–follower values contributes to adverse outcomes. Much research has demonstrated the value of coherence between the values of staff and organisations [9]. The relevance of the leader’s values being in harmony with those of other organisational members—such as peers, supervisors, interviewers, and working groups, as well as the organisation itself—from a practical and scientific perspective, is known as value congruence [10]. Since value congruence can result in several beneficial effects, such alignment has been found to be an effective strategy to persuade individuals within businesses.

At the time that this study was conducted, there had been no published study carried out in Saudi Arabia exploring the value congruence between nurse leaders and their staff, whether subjective or objective. According to Hiller et al. (2011) [11], there has not been enough research carried out on the concept of value congruence—particularly with regard to leadership style and how it affects leader–follower value congruence in successful firms [12]. In Saudi Arabia, almost all of the tertiary hospitals have a heterogeneous health workforce composed of different nationalities with varied languages, cultures, religions, and even values; hence, the merit of exploring the value congruence among the nurse leaders (NLs) and the staff nurses (SNs) in one of the biggest tertiary hospitals in Saudi Arabia is deemed worthy of pursuit.

## 2. Materials and Methods

### 2.1. Design

This study employed sequential mixed methods including descriptive qualitative and quantitative approaches (Table 1). The exploratory sequential design began with the gathering and analysis of qualitative information, and then progressed to the collection and analysis of quantitative data, followed by interpretation. The results from the interviews in the qualitative phase were used to create a new instrument or taxonomy for the quantitative phase of the study [13]. The exploratory sequential method was deemed suitable for this study as it explored congruent values, which had not yet been established in the institution of interest. The number of nursing service staff is huge; thus, a qualitative part to establish the construct for leadership values was imperative. These themes or constructs were then used in developing the tools to be used in the quantitative phase.

#### 2.1.1. Phase 1: Qualitative Design

Descriptive qualitative research through interviews was conducted among 33 nurse leaders and 37 staff nurses to determine the perceived effective leadership values of nurse leaders. An interview protocol was developed and followed to ensure consistency. The interview protocol included the following: The grand tour question was pilot tested among the researchers before a second round of testing for the staff nurses and nurse leaders to ensure that the questions were neutral and culture-sensitive. The duration of the interview was set from 5 to 10 min, with English as the language, and the venue was established considering the safety and privacy of the participants. A brief introductory meeting was set with every participant before the scheduled interview, so as to ensure comfort and ease between the interviewer and the participant. Consent included orientation with respect to the purpose of the research and their participation, recording of the interview, and publication of the findings of the study. Reflexive bracketing was performed prior to the interviews to control any biases from the interviewers. The questions asked in the interviews were semi-structured, starting with the grand tour questions *“What are your leadership values as a nurse leader that you consider most important to ensure teamwork with your staff nurses?”* and *“What are the leadership values of your nurse leader that you consider very important to you as a staff nurse?”* Follow-up questions were based on the answers of the participants. The interview transcripts were transcribed and reviewed [14]. Purposive sampling, which is the established sampling technique for qualitative designs [15], was used to select the participants among the nurse leaders and staff nurses.

#### 2.1.2. Phase 2: Descriptive Quantitative Design

The descriptive quantitative approach, which was the second phase of the study, determined the perceived effective values of nurse leaders from the larger group of nurse leaders and staff nurses from the different hospitals of the medical city. The data were collected from 105 nurse leaders and 466 staff nurses. Purposive sampling was used with the following specifications: (1) Nurse leaders and staff nurses should have been in their current positions for at least a year. (2) Nurse leaders and staff nurses should have worked together in the same location. (3) All of the staff nurses and nursing supervisors at the multicultural organisation were from one of the top five most prevalent nationalities. (4) Participants must be able to communicate in English. Since the nationality of the nurses was diverse, stratified sampling was carried out to ensure that every nationality was properly represented. Stratified sampling includes subjects from every subgroup, ensuring that the sample reflects the diversity of the population [16]. 

#### 2.1.3. Questionnaire

The effective leadership values identified from the interviews in the qualitative phase of the study were transformed into a quantitative questionnaire to be used in the second phase of the study. This questionnaire underwent construct validation from expert nurse leaders and staff nurses, as well as university professors. A reliability test was carried out before it was used in the second phase. The Cronbach’s alpha yielded a value of 0.70, which indicates a good level of internal consistency.

The questionnaire was composed of two parts: the first part consisted of questions on the sociodemographic profile of the respondents, while the second part was composed of 62 items related to the leadership values of the nurse leaders. These 62 questions were categorised into six groups corresponding to the six themes that emerged from the qualitative phase. Each category included questions related to the subthemes. The questions were answered on a Likert scale of 1–4.

### 2.2. Ethical Considerations

Ethical approval from the selected hospitals (ref No. H-01-R-012 and ref ERC No. 2020-049-IGS-CNA) was granted. Administrative clearance was obtained from the hospitals, and consent was provided by each participant after the research team explained the purpose of the study and the expectations from their participation, including privacy and confidentiality. 

### 2.3. Data Analysis

The emerging themes were developed following the seven steps of qualitative data analysis described by Colaizzi [17]. The interview transcripts were read several times to ensure familiarity with the text and its meaning. After appraisal of the transcripts, we identified significant statements that were relevant to the phenomenon being investigated. Meanings were formulated from the significant statements, the formulated meanings were clustered into themes, exhaustive descriptions were written to capture the structure of the phenomena and, finally, the formulated themes were shown back to the participants to confirm that the correct context was captured. A secondary reviewer from the university, in addition to one of the co-authors, was involved to ensure the correct interpretation and analysis of the verbalisations and emerging themes. The final themes were reviewed, and modifications were implemented when necessary.

For the quantitative phase, the values considered as necessary from a nurse leader were determined through the mean scores of the different leadership value scales. ANOVA was used to determine variance between the staff nurses’ and nurse leaders’ perceived leadership values. Significance was set to p-value less than or equal to 0.05.

## 3. Results

### 3.1. Sociodemographic Profile of the Participants in the Qualitative Phase (n = 70)

There were 70 participants in the qualitative phase of the study. The top five (5) countries of origin in the multicultural organisation were the Philippines, India, Saudi Arabia, Jordan, and South Africa. As expected, there were more females compared to males, considering that the nursing profession is dominated by women (Table 2).

### 3.2. Sociodemographic Profile of the Participants in the Qualitative Phase 

The top respondent for this study was the rehabilitation hospital (23.3%), followed by the women’s specialised hospital (19.3%) and the main hospital (13.3%). The majority of the respondents were in the age bracket of 31–38 years (46.6%), followed by 23–30 years (22.1%) and 39–46 years (17.7%). Most of the participants were from Asian countries (92.29%), followed by countries in the Greater Middle East (6.13%) (Table 3).

### 3.3. Congruent Values among the Nurse Leaders and Staff Nurses

**Figure 1:**
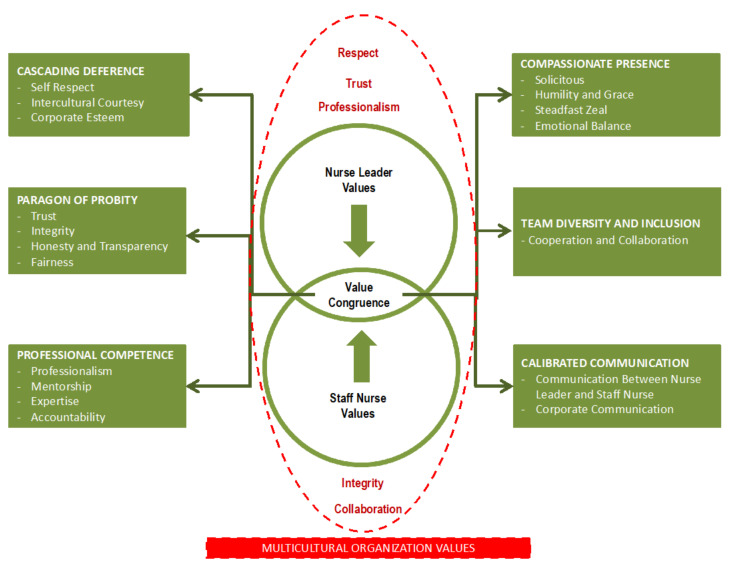
Themes and subthemes: congruent leadership values among the nurse leaders and staff nurses.

The result of the interviews performed among the nurse leaders and staff nurses, in terms of what are considered effective values that a nurse leader must have, were coded and analysed, bringing forth the following themes and subthemes (Figure 1):

#### 3.3.1. Theme 1: Cascading Deference

Cascading deference is a distinct moral duty for every individual to have positive attitudes toward human beings’ worth, concerns, and dignity, regardless of race, culture, or religion. Among the values expressed in this theme are respect, intercultural courtesy, and corporate esteem. The following experiences were described by the nurses, highlighted in the various subthemes. Note that NL indicates nurse leaders and SN indicates staff nurses.

##### Subtheme 1.1: Self-Respect

Self-respect as knowing oneself, including one’s identity, feelings, abilities, and value.

*NL #04: “As Nurse leader I should start respecting myself so that I am also worthy of the respect from other people. Self-respect can prevent others to disrespect you or treat you bad because when you display your ability to show who you are professionally; they see the confidence of a great nursing leader”.*

*NL #05: “Self-respect is when I accept of who I am and what I can do things. I have to check and be aware of my capabilities so that I can use in helping myself and my staff in certain situations”.*

*NL #14: “Respecting each individual weakness including self should be always exercise as we learn and rise above from our weakness to know your strength which will make a one leader firm in setting boundaries”.*

*SN #04: “Nurse leaders should have self-respect because having this value will give them the unique identity as a nurse leader where they believe on their capacity and what they can do for the staff nurse and department”.*

*RN #19: “They (nurse leaders) should have self-respect where they are confident with their capabilities as a leader where they can independently have arrived solid clinical judgement to every situation that can arise in the area. If they have self-respect the respect to the subordinates will follow regardless of different position and nationality. By respecting yourself, people around you will also create a respectful environment where everyone succeeds in this kind of workplace”.*

*SN #30: “My line manager has high respect to his self where he displays confidence with his judgement. He always backs up with policies and procedures to explain such judgement. So, I salute this kind of leader in our institution”.*

*SN #33: “Respect can be demonstrated by either self-respect or respecting others dignity regardless of differences. Self-Respect is when they decide to be honest with themselves and recognize their leadership abilities. We can see nurse leaders who have high respect to themselves because it exudes their confidence, and they are very firm with their decisions”.*

##### Subtheme 1.2: Intercultural Courtesy

Intercultural courtesy was defined by the participants as respecting each individual person in terms of their own religion, culture, and norms. The statements indicate that the nurses respect the cultural beliefs of patients and other members of the healthcare team. This shows that efficient communication within the intercultural team is present and that high-quality, safe patient care is provided. Nurses must become more conscious of their own cultural views as well as the cultural elements that influence intercultural courtesy.

*NLs #1, 2, 6, and 26: “I have to make sure that my team feels respected at all times”.*

*NL #14: “Respect should be their main value in making a decision that concerns staff nurse regardless of their race, culture and colour and as a leader, we must respect others culture by giving them a chance to celebrate special holidays like Ramadan, Christmas and Diwali”.*

*NL #20: “Her consideration during Ramadan meals at staff lounge is highly discouraged as possible to pay respect to the Muslim staff who are on fasting and respect the Muslim by giving them time to pray called ‘Salah’”.*

*NL #30: “SSSHHH to stop talking in my culture because it’s not accepted due to we use this to animals. I don’t trust leaders who is not good in respecting others culture”.*

*SN #10: “Her nurse leader makes sure that she treats everyone with respect and is fair to everyone regardless of race and nationality”.*

*SN #12: “Our nurse leader shows teamwork by respecting everyone (Nurses, Physicians, Allied Healthcare, patients, and Family) at the workplace. She also added that nurse leaders should make sure that all the practice, belief and culture is being considered”.*

*SN #13: “A respectful a true leader must show a humbling and admiring attitude by respecting all workmates regardless of culture, language and any personal performances and differences”.*

*SN #18: “Respecting each person, the same regardless of nationality and culture is the ultimate values of nurse leader must one possess in the unit”.*

##### Subtheme 1.3: Corporate Esteem

According to this study, corporate esteem is defined as having one’s opinion, views, rights, feelings, and wishes respected at work. Based on the nurses’ verbalisations, we can glean that nursing leaders can model behaviours that build mutual trust, respect, and competence. This suggests that the nursing leaders’ values encourage and inspire proper behaviour.

*NL #10: “I practice respect even if my subordinates commit mistakes. I immediately call their attention and sit with them in the right venue… as I believe that reprimanding the staff respectfully for their wrongdoing will help them grow their professional career”.*

*NL #11: “Staff nurse should also respect the decision of nurse leaders in terms of allocation, should always follow the chain of command and do not practice bullying in the unit”.*

*NL #18: “Respecting the opinions of each other is a great value of a nurse leader to make things lighter and smoother among nurses”.*

*NL #23: “As a nurse leader, respecting the staff nurse to have their own suggestion, opinions and ideas at work will help the nursing leaders do their job properly as I believe Respect begets respect”.*

*SN #03: “When problems occur in our unit, she (Unit Manager) takes immediate action into it in a very respectful manner and decides what is the best for the staff, family, or organization”.*

*SN #17: “Respecting positive and negative responses alone is a value they must possess at the workplace as they deal with many people. The nurse leaders are practising this in a monthly unit meeting where everyone has the chance to voice out concerns and where they expected to arrive at a sound decision for the improvement of nursing services”.*

*SN #20: “A nurse leader should be respectful by hearing out opinions and suggestions from staff nurse without developing fear or judgement…they respect you don’t have to be limited within the mandate of the workplace but instead they will see you as additional input in the unit”.*

*SN #23: “Respecting the rights, wishes and feelings of others during handover, meeting on any discussion held at the ward”.*

#### 3.3.2. Theme 2: Paragon of Probity

Paragon of probity is defined in this study as the idea that a nurse leader should be able to encourage and ensure proper ethical conduct in their activities and interactions in the workplace, and they should use ethics to lift staff morale and make their subordinates feel fair and enthusiastic about their leadership and their work. Trust, integrity, honesty, and fairness are among the values mentioned.

This theme shows the situations—particularly in terms of nurse leaders’ roles—that foster work engagement of nurses in hospitals, which is vital to the organisation. 

##### Subtheme 2.1: Trust 

This study defines trust as maintaining patient and staff privacy and confidentiality, as well as putting one’s words into action. As presented, the nurses exemplify trust in their daily routine. They demonstrate trust and trustworthiness when dealing with their patients, their superiors, or their subordinates. This suggests that the nurse leaders have faith in their staff and, therefore, the staff are more likely to engage in their work if they are called upon to do their duties.

*NL #02: “Trust is a very important value among staff, patient and healthcare workers to keep the workflow and increase work productivity”.*

*NL #09: “Trust enhances teamwork, and collaboration and enhances decision making at the workplace where it—it empowers the staff to make their decision and to have the confidence and courage”.*

*NL #21: “Trustworthiness is to maintain the staff integrity and behaviour. The trust in all the actions of the nurse leader will improve the working environment”.*

*SN #2: “Trust is given when all conversations with the leaders are treated confidentially”.*

*SN #5: “A Trustworthy nurse leader can demonstrate privacy by maintaining patient confidentiality by keeping all records, locked, allowing access only to people with a need to see information about patients and staff”.*

*SN #7: “A trustworthy nurse leader are expected to be helpful to the staff as they depend on him on everything (clinically and operational). She cites an example of trust where staff should trust you with all your clinical decisions as use the policies and procedures as basis for our caring our patient”.*

*SN #16: “If nurses are unsure with anything, they will approach the nurse leader in the unit who can always show guidance at any point of the shift. When a Nurse leader give the right direction and help the staff nurse solve problems, it makes them easy to trust this kind of nursing leader”.*

*SN #27: “A Trustful leader is when they will be able to help you with problems and give you feedback in return and Trust can build faster decision making in the unit”.*

*SN #36: “Nurses are the most trusted group of people in the healthcare setting and a culture of trust is the most essential nursing leader values as it increases commitment to the team goals as they become more comfortable with change and more willing to embrace”.*

##### Subtheme 2.2: Integrity 

According to this study, integrity is the capacity to form moral judgments and to act morally even when no one is watching. As stated in the nurses’ articulations, a sense of moral integrity offers a certain degree of consistency that can assist the nurses in dealing with uncertainty. This indicates that the nurses trust ethical nurse leaders because of the behaviour that they exemplify by being credible and trustworthy.

*NL #3: “Integrity is to know and to do what is right and to display our best behaviour, being honest and practice self-control in difficult situations”.*

*NL #16: “Firmly believed that Integrity to know and to do what is the right thing even when nobody is looking or listening because we are dealing with patients’ lives and if integrity is not practiced it will have negative impact in patients care. She further explains that “not having the time to check blood sugar will result to wrong medication dose impact outcome”.*

*NL #22: “Our actions reveal our commitment in telling the truth and how we always see ourselves from perspective of others”.*

*NL #26: “The value of Integrity is reflected in our professional practice as nurse leader, for example when the nurse leader is honest and provides sound decision based on their own ethical framework that they acquired through the years”.*

*SN #9: “Integrity is when nurse leader is capable to make moral decision following policy and at the same time making effort to protect their staff. Nurse leaders never discriminates any staff and would always show fairness when it comes to decision making merits, acknowledgement and reprimand the wrong one without considering personal relationship such as being a friend or ally”.*

*SN #21: “Integrity reflects in professional practice when nurse leader is honest and provides care based on ethical framework; whenever rendering care to patient, we should render care with or without anyone watching us”.*

*SN #23: “Integrity of the nurse leaders should be genuine and honest where they decide to do the right thing at all-time especially if there is urgent problem that arise, we staff count on them”.*

*SN #26: “Integrity is when you need to document truthfully series of events maintaining the integrity of the nursing professions where you put aside your personal opinions as nurse leader”.*

##### Subtheme 2.3: Honesty/Transparency

This study defines honesty and transparency as being truthful and disclosing the rationale for all actions and decisions undertaken. The accounts indicated that the nurses must share the value of fostering a culture that supports patient safety. This signifies that the nurse leaders can create an atmosphere in which every team member feels a duty and is responsible for making sure that the goal of keeping patients safe is upheld.

*NL #04: “Honesty should start with myself as nurse leader; I should be honest with myself by accepting task that I know I can do. If a task is assigned to me and I knew I am not capable of doing, I should be honest enough to tell that I cannot do or cope up with the task assigned”.*

*NL #06: “Honesty of the nursing leaders is a value they must have in multinational environment where nurses are often concerns to allocation, benefits, request schedule, leave planner and shift in charge role and we should always be honest and transparent when we are preparing to build confidence in your leadership. Being an honest nurse leader build trust within you team and I practice this by giving the correct and updated information about patient, policies, and goals to all my staff and colleagues”.*

*NL #25: “I practice honesty when I think I have gaps this is when I encounter things that I don’t know a certain procedure, I will not pretend to know everything as we are dealing with life of people, any mistakes will cause their life”.*

*SN #03: “If we did something wrong, nurse leader should be honest enough to give constructive criticism for us to improve”.*

*SN #04: “Transparent nurse leader can accept new ideas, provide equal opportunities to staff, patient and organization to improve the nursing care”.*

*SN #09: “Transparency of all information including plans (unless sensitive issues or confidential matter) must be relayed to nursing staff”.*

*SN #11: “Honesty is always telling the truth and giving honest evaluation of what truly transpire in certain events such as meetings, yearly evaluations and why they derive that decision. Transparency can be observed during annual evaluation where nurse leaders show us why she gave that evaluation score”.*

*SN #16: “Honesty means nothing showed under the rug, if any lacking in the documentation, nurse leader should immediately correct the gap”.*

*SN #22: “Having a transparent nursing leader can improve the safety of the patient and staff as the nurse leader can get additional input/ideas operationally from the front liners”.*

*SN #23: “Honest discussion of topics or issues with colleagues in a clear and fruitful manner during handover to avoid errors and miscommunication must be upheld by nurse leaders”.*

##### Subtheme 2.4: Fairness

Being fair is defined as treating every employee equally and applying the rules to everyone. The accounts indicated that the nurses embody fairness and equality in their practice with their patients and other members of the healthcare team. This suggests that the nurses do their best to drive changes in patient care that protect the humanity, equality, fairness, and dignity of all patients and different members of the health team.

*NL #4: “Being fair and equal to all nurses is a must as it cuts conflicts and increase trust in workplace”.*

*NL #18: “As nurse leaders we (nurse leaders) should try to treat the staff equally by giving opportunities to the staff to participate the group activities and unit routines and arrange team building activities and make them to participate as monthly”.*

*NL #22: “I need to have different approach in dealing with my staff nurse and I believed that social justice promotes fairness and non-discrimination environment to different nationalities because you have to see them equally”.*

*NL #30: “Nurse leader promote justice and fairness in terms of our decision making and hearing all side of the story should be considered to see the pros and cons of our action”.*

*SN #03: “That a fair nurse leader treats staff nurse equally regardless of nationality”.*

*SN #06: “Fairness is giving staff their request especially on scheduling on scheduling on vacation. This motivates the staff and trust their leader”.*

*SN #13: “He/ she is the captain of the ship hence must treat all equally and with fairness. Example: a leader must communicate and deal with all nurses at the same level with he is treating with all nationalities equally and fairly in terms of schedule, vacation request or in times of emergency”.*

*SN #14: “The value of fairness is important in delegating task and responsibilities of staff nurses”.*

*SN #19: “I admire my nurse leader who is taking care of everyone by being fair, no discrimination, accepting limitation of all, and resolving conflicts within the unit immediately”.*

*SN #22: “my nurse leader is trying his best to be fair to all his staff in terms of his/her decisions or actions. In line of those noted that her nurse leader is fair because he is basing his disciplinary actions from the organizational policies”.*

*SN #29: “She is working with us observing equality despite of different culture and language”.*

#### 3.3.3. Theme 3: Professional Competence

Collectively, this study defines professional competence as possessing the knowledge, competence, or skills necessary to achieve something in managing staff nurses. One nurse leader is anticipated to be able to apply their talents and expertise to address problems in their respective field. This knowledge, talent, or skill can be acquired via formal education, training, and/or experience. Professionalism, mentorship, expertise, and accountability are collective values that are part of professional competence.

Moreover, this theme shows the incidence of nurses’ sets of abilities, attitudes, beliefs, and knowledge that contribute to effective or high performance in positions of employment and professional standing. 

##### Subtheme 3.1: Professionalism

According to this study, professionalism entails taking ownership of problems and challenges as they emerge and handling them in the most dedicated way possible. As specified in the nurses’ verbalisations, the nurses believe in providing patients with high-quality treatment while respecting the principles of responsibility, respect, and integrity. This implies that the nurse leaders are offering the ideal environment for professional development in clinical practice.

*NL #1: “I practice Professionalism by doing my best to execute all the responsibilities assigned to me”.*

*NL #2: Professionalism and productivity at work is one value I always practice by making sure that the unit, staff, and patient is functioning at their best”.*

*NL #08: “I exhibit professional behaviour at all times by completing my projects as soon as possible to avoid piling up of incomplete projects”.*

*NL #09: “Professionalism is meeting the deadline is a great value by great leader and team leader”.*

*NL #13: “Professionalism is one of the most important values that a leader or all nursing should have. By being role model in terms of dressing and acting appropriately so staff will also reflect the same professionalism as the nurse leader All actions that you have to do should be done with professionalism. Professionalism for me is when all decisions or things you do will benefit other people especially with patients”.*

*SN #03: “Professionalism is being knowledgeable in whatever problems will occur, conscientious in her actions or decisions to make and responsible for her subordinates”.*

*SN #5: “Professionalism is to stay in a profession and sense of responsibility towards professional problems and challenge where nurse leaders can help to provide an optimal patient care promotion the nursing profession”.*

*SN #20: “Nurse leader should be professional at all times and to make decision not based on person feelings but for the good of the unit or staff”.*

*SN #24: “Professionalism is to separate individual agenda where work is work, friends are friends”.*

*SN #26: “Nurse leaders must protect a client privacy, design care and sensitivity to individual client needs and acts accordance with the code of ethic and accepted standard to practice”.*

##### Subtheme 3.2: Mentorship

This study defines mentoring as having the capacity to guide and uplift others toward achievement in their professional or personal development. The verbalisations indicate that the nurses have collaborative partnerships between the nurse leaders and staff nurses. This shows that in the increasingly demanding and stressful healthcare workplace, mentoring has proven to be a successful technique for developing nurses.

*NL #07: “Mentor staff to achieved certain goals. For example: Leading the staff nurses to make sure that every order (procedure, diagnostic and treatment) is carried out. All patients should be vitally stable, and the leader should establish a good communication within the multidisciplinary team should be properly established”.*

*NL #08: “The challenge of being a mentor is where I always update my knowledge with new developments in my field by reading or studying as I believe that when I increase my professional growth, I can do more with my staff concerns”.*

*NL #11: “Mentorship is one value that a leader must possess. This value will maximize the potentials of every staff nurse and it improve our overall work performance too”.*

*NL #12: Mentor staff nurse to grow professionally and able to inspire them to engage in our mission, vision, and goals in our organization”.*

*NL #17: The value of mentoring the staff for any task till it will be executed well and up to standard. When you motivate the staff, they do even better with patient care and the unit objectives”.*

*NL #20: I believe that my values to motivate and mentor staff nurse who demonstrate willingness to learn and ready to accept criticism for them to improve gives them a lot of change to grow as effective staff nurse and future leader”.*

*NL #23: “We are all professionals and we have attained the acquired skills and knowledge by our education and practice”. She further explains her point of view “A true value of a leader should be reflected to his knowledge and skills doing things the right way according to evidence-based practice, policies and procedures”.*

*NL #26: “Sharing of knowledge is a wonderful value of a leader because when you mentor people, you see them excel in the field of nursing”.*

*SN #01: “One values of the Nurse leader must have is being mentor by being at the bedside in times of critical moments like code blue, accreditation and dealing with patient and family problem”.*

*SN #6: “A good nurse leader is a mentor where they set a good example for following the hospitals rules and regulation.”*

*SN #30: “A nurse leader who mentors listen to all concerns of the staff, give them a good advice and solutions to the problems. staff was treated as a family member which work hand in hand to achieve our goal in the unit that is render quality care for all the patients”*.

##### Subtheme 3.3: Expertise

This study defines expertise as demonstrating a profound understanding of their field through training, experience, and formal education. The nurses’ descriptions showed the nurses gain expertise when they put their theoretical and practical knowledge to the test in real-world clinical settings and refine their clinical judgment and level of care. This implies that the nurses set themselves apart from their peers by frequently showing an intuitive capacity to effectively make crucial clinical decisions while understanding the full context of a scenario.

*NL #2: “An ideal leader exhibits a deep sense of knowledge about their work through advance studies in leadership and management”.*

*NL #08: “I always update my knowledge with new developments in my field by reading or studying as I believe that when I increase my professional growth, I can do more with my staff concerns”.*

*NL #16: “Expertise with changes in management and practice also leads to better patient outcomes and improves quality care. It allows staff to increase their skills level and apply theory to practice, this also leads to patient and staff satisfaction improvement”.*

*SN #05: “A nurse leader must have sense of autonomy by being knowledgeable and confident to make independent decision about clinical practice”.*

*SN #07: “Nurse leaders should be knowledgeable and competent where they should know how to run a unit, how to manage staff and knows the policies and procedure of the organization”.*

*SN #20: “A good nurse leader should have a good critical thinking in dealing with situation where they are expected to act accordingly to promote safe nursing practice in the area”.*

##### Subtheme 3.4: Accountability

According to this study, accountability is defined as taking ownership of one’s deeds, choices, and obligations as a team player and leader. The nurses’ reports show that the nurses are willing to bear accountability when and if there are breaches of patient care, in addition to having high standards for their own clinical practice and ethics. This indicates that the nurses are responsible for developing their clinical skillsets and adopting the highest-quality results of evidence-based practice to inform their nursing treatments and accommodate the increasing healthcare demands of patients who are living longer with chronic illnesses and complex disease processes.

*NL #05: “I strongly believed in being accountable in our actions and responsibilities so that staff can trust you with your decisions”.*

*NL #14: “Nurse leader must practice accountability and professionalism—as a person and a leader you must possess value of accountability in what you are doing. As a charge nurse in my unit, I make sure that my staff performing correct procedures follow hospital policy and procedures”.*

*NL #23: “Accountability—by mean of being accountable or responsible with all your decisions in the unit. You should be able to do a sound decision for the best interest of everyone”.*

*NL #24: “As nurse leader, I must be clear about our own professional role and responsibilities in our organizational goals. I must provide direction to all my staff nurses on how we can work together on to be able to be accomplished designated task. In this manner we can build a culture of accountability”.*

*SN #06 and RN #37: “Accountability is where nurse leader is being “responsible for all his/her own decisions”.*

*SN #12: “Nurse leaders should always uphold the integrity of the profession by being responsible for their own action towards greater good for the staff, patient and organization”.*

*SN #13: “A good leader must possess the sense of accountability not only as the head of the group but as member of the team where they set a good example to the team by owning his decisions and responsibilities”.*

*SN #26: “Accountability of nurse leaders means have the right power and competence to evaluate client care and implement changes in health care practice to improve outcomes within the health care system”.*

*RN #36: ”Accountability in healthcare setting, this value has become as significant concern for nurse leader where everyone must practice into their actions and plans. It also empowers the value of patient care. As nurse leader be clear about professional role and responsibilities and organizational goals. An accountable nurse leader provide direction about how work should be accomplished and build a culture of accountability”.*

#### 3.3.4. Theme 4: Compassionate Presence

The term “compassionate presence” is defined as an attitude toward all people that involves self-balance and passion, with a focus on showing compassion and empathy for those who are in need in the multicultural organisation. Solicitousness, humility and grace, steadfast zeal, and emotional balance are the collective values mentioned in this compassionate presence theme. 

##### Subtheme 4.1: Solicitousness

In this study, the term solicitousness refers to the ability to relate to other staff members’ situations with empathy and compassion. The nurses stated that they practice compassion every day. This demonstrates that the nurses embody this fundamental part of the very ethos of nursing and, consequently, achieve improvements in staff satisfaction, patient health outcomes, and patient satisfaction with care.

*NL #01: “A compassionate leader is a value of nursing leader who understand his staff/patient welfare making sure that all things are deriving a decision”.*

*NL #04: “I always listen to my staff and try to understand their feelings and situations they are faced with even If I know that a leader, I cannot satisfy all the staff, but I try wherever and whenever possible to satisfy staff as long as it is not against the hospital policies and not compromising patient care. Being Emphatic nursing leader is where I acknowledge the fact that we are all human with human flaws where everyone must acknowledge their wrongdoing and take it as learning opportunities”.*

*NL #09: “The value of compassion is executed when I must understand the staff sufferings in some situation where I am always ready to give time to listen so that the staff will understand and feel that there not alone in their battle”.*

*NL #12: “Empathy is by showing concern and support for colleagues such new staff in every way possible to help them perform and grow as it improves communication, relationship, and processes. Nurse leader who uses the value of empathy has the ability to understand the feelings and emotions of others and respond to their action by listening and understanding their concerns”.*

*NL #24: “Compassion is a value that promotes fairness and avoid favouritism as we put ourselves in their shoes to understand them more. A nurse leader must use the value empathy where I should be open to all staff where they can ventilate their concern and clarifications”.*

*SN #02: “Most of our charge nurses are compassionate to us because they empathize in times of toxic duty where if they come and help with pending task or procedure”.*

*SN #05: “One of the fundamental values of nursing leader is to be compassionate where they exercise to be empathic to patient dignity and patient family needs”.*

*SN #26: “Compassionate leader is being concern of the wellbeing to others; being able to understand others culture, beliefs., perspective and also help to motivate others and work without reward and recognition”.*

*SN #36: “Compassion is the foundation of nursing value because they can pay a significant role in supporting staff and for the improvement of working environment”.*

##### Subtheme 4.2: Humility and Gratitude

According to this study, humility and gratitude entails the acknowledgment of another person’s growth and accomplishments while putting aside pride and ego and being modest. The nursing leaders can foster an environment where the staff’s dispositional humility grows and their own character flourishes. This implies that the nurses are willing to learn from others, do not flaunt their status or accomplishments excessively, express gratitude to others, and are able to apologise for missteps.

*NL #3: “If you are successful or not you have to show gratitude to all handworks and contribution of all the staff nurses”.*

*NL #11: “Gratitude is shown when I compliment my team daily for always working hard, taking care of our patients, and keeping our unit well organized, neat and clean. I also encourage each of them to say, ‘thank you’ to their colleagues, actually made a shout out board for them to post simple notes of gratitude as this helps reduced stress and motivates them to work smarter”.*

*NL #25: “Humility and gratitude is a tandem of values where I usually weigh things that are suggested and compare them to my strategies and if I think their ideas are more applicable and sensible, I give my utmost gratitude by crediting the work to them. Leader who shows gratitude to all opinions and suggestion of the staff nurse has also shown humility by accepting constructive criticism”.*

*SN #3: “A nurse leader should be humble with their achievements, and this will create a non-judgmental environment”.*

*SN #20: “A good nurse leader should be humble and exercise humility where they can acknowledge their own mistakes and willing to listen to suggestion and criticism without taking any personal grudge to the staff nurse”.*

*SN #21: “Humility is a quality or state of being humble where they should never look down or treat us with arrogance, because his/her effectiveness as leader reflect on us”.*

*SN #32: “Nurse leaders who practice humility equates to being humble and setting aside your pride and ego”.*

##### Subtheme 4.3: Steadfast Zeal

In this study, steadfast zeal is defined as setting an example for others by making extra efforts to raise everyone’s standard of living. Nurses can experience the satisfaction of helping others while developing both professionally and personally, as stated in the accounts above. This indicates that the nurses experience a strong proclivity for activities that they enjoy, find significant, and devote time and effort to.

*NL #05: “A Passionate leader who encourage the upper management to give promotion, vacation, salary increment. Gives promotion to deserving staff nurses without having the bias of our personal insight, Giving enough vacation to all staff equally as they are expats they need to be with their family as they are away from them for long time. Gives the right and fair evaluation as it affects their salary increase”.*

*NL #27: “Passion to serve the staff patient and my hospital- I am always willing to go extra mile and learn new things for improvement… striving to be good in yourself and for others”.*

*NL #31: “A nurse leader who is a passionate leader is the one who is not only looking at the goals of the organization but also see what he/ she can help to improve the staff welfare”.*

*SN #08: “A nurse leader who is passionate will persistently reinforces results not only to the unit goals but also our personal goals”.*

*SN #32 “Passionate, optimism and enthusiasm are a great nurse leader values that inspires others by keeping genuine keenness, passion and their zeal for what they do”.*

*SN #33: “A passionate leader must genuinely care about their work where they create a culture where the team members feel inspired and become passionate to their work which can results to higher productivity of work”.*

##### Subtheme 4.4: Emotional Balance

This study defines emotional balance as the capacity to experience emotions in the face of adversity while still employing logic to come up with solutions. Nurses have the capacity to control their own emotions as well as those of others. This enables the nurses to make wiser choices, manage their patients better, build stronger bonds with patients and families, and strengthen relationships.

*NL #29: “The value of emotional intelligence is seen where nurse leader deals with their self-emotions when facing critical situation and/ or during high emotions. This will help the nurse leader to separate emotions and being rational which will eventually solve any problems that has been encountered”.*

*NL #33: “Emotional intelligence is used by nurse leader to support the colleague to cope up with their stress and managing their challenges”.*

*SN #10: “A nurse leader must emotional intelligence where they are equipped to support nursing staff in coping up with different stressors at the workplace. Having a great emotional intelligence will have the sense of security what emotions should be display to certain events”.*

*SN #17: “The courage to understand situations by using their own value emotional intelligence and wisdom is a must in working with a multicultural workplace as this will help them dwell with different cultural differences of their staff nurses and they know which hat of emotions to put on at the right time”.*

*SN #25: “Emotional Intelligence is crucial for nurse leader in multicultural healthcare organization*

*Where these values can help them understand each other differences, perspective, style of thinking and different disciplines of the health professionals”.*

*SN #34: “Nurse leader who has emotional intelligence uses self-awareness to recognize one’s ability and make boundaries to his her/ assignment, the nurse leader who knows how to use certain emotions or values has a great sense of emotional balance”.*

#### 3.3.5. Theme 5: Team Diversity and Inclusion

This study defines team diversity and inclusion as a group’s cooperative effort to complete a task successfully and efficiently or to accomplish a common goal. Knowing each function and responsibility and utilising them effectively for increased productivity helps to further develop this idea. Collaboration and cooperation are shared values that are part of the team’s diversity and inclusiveness.

Furthermore, this theme shows the incidence of nurses’ teamwork, where a variety of elements influence team performance, with team leadership possibly being one of the most important.

##### Subtheme 5.1: Collaboration and Cooperation 

In this study, collaboration and cooperation are defined as knowing one another’s (i.e., the nurse leader and staff nurses) strengths and using them to the team’s benefit to achieve a certain goal. The nurses have high levels of teamwork and collaboration skills. This shows that the nurse leaders are there when the nurses need expert assistance with technical tasks, thereby providing patients with safe, high-quality care.

*NL #07: “Teamwork is seen during code blue activation; we as single unit tend to focus on one task and that is keep patient safety”.*

*NL #09: “Enhancing teamwork and collaboration in the workplace has a big impact on how staff collaborate and work together in decision making”.*

*NL #19: “Teamwork is when they are giving a helping hand during code blue incident”.*

*NL #21 “Collaboration and teamwork should go hand in hand in workplace to have a successful shift where you accomplish all the required nursing care and goals”.*

*NL #23: “There are times that we can expect toxic days, but if all staff nurse helps each other burden will be lessen and allow all to them to go home on time”.*

*SN #01: “Nurse leader with good leadership will collaborate with their staff and give extra support to for the general welfare of the staff nurse”.*

*SN #31: “The nurse leaders exercise teamwork when they understand the capability and needs of their staff by promoting individual strengths to work efficiently”.*

*SN #32: “Nurse leader should focus in creating effective teamwork by promoting team building activities where this fosters camaraderie between staff nurses and nurse leaders”.*

#### 3.3.6. Theme 6: Calibrated Communication

In this study, calibrated communication is defined as being aware of the communication flow between two parties, with the ultimate objective of reducing communication barriers. The nurses shared the following descriptions underscoring the various subthemes:

##### Subtheme 6.1: Communication between Nurse leader and Staff Nurse 

This study defines communication between nurse leaders and staff nurses as occasions in which nurse leaders and staff nurses successfully communicate across cultural boundaries as a healthcare team. The nurses who participated in the interviews all agreed on the significance and necessity of effective communication, both in the working relationship with the patients and in the relationship with the nurse leader. These findings imply that nursing leaders’ instructional leadership might enhance performance and help the hospital reach their goals.

*NL #12: “Minimize the language barrier and maintain etiquette because many cultures have specific etiquette on how they can communicate, so we have to speak one language, speak slowly, and simple to understood each other”.*

*NL #15: “I communicate with all staff nurse on a level where I show interest—not just interest but real interest by active listening and discussion with their concerns”.*

*NL #22: “Value of communication between nurse leader and staff nurse should be clear and let them speak up for themselves to hear their side of the story whenever there are conflicts or suggestion being raised”.*

*NL #29: “Working with different nationalities would be challenging but a leader must work with an open communication and fair go to all. An equal and direct interaction to all level of nursing staff is very important to discuss agenda of the unit”.*

*SN #17: “Being aware of how to communicate and convey the message to staff nurses is a great value of a nurse leader”.*

*SN #01: “The value of being good listener is seen in a nurse leader when she listens and accept new ideas during huddle or meetings”.*

*SN #04: “A nurse leader who communicates effectively his plans to all staff nurses increase discussion between nurse leader and staff nurses leading to more productivity in the unit”.*

*SN #10: “A nurse leader should have a good communication skill to facilitate a good collaboration between multidisciplinary team”.*

*SN #18: “Communication skills is excellent trait of a nurse leader where they can easily express their thoughts and ideas to all staff where it helps to improve the exchanges of ideas between staff and leader”.*

*SN #13: “Nurse leader must know the importance of verbal and nonverbal communication and effective cross-cultural communication to address concern or response building and maintaining non-judgmental”.*

*RN #30: “A good leader must be a good listener, they listen to all concerns of the staff, give a good advice and solutions to the problems”.*

*RN #16: “Our Nurse Leader exercise frequently communication to all staff giving the right direction or goals thus, this makes it easy to trust her because she doesn’t single out a person in disseminating information”.*

##### Subtheme 6.2: Corporate Communication

In line with the findings of this study, corporate communication is defined as the use of a communication method that is permitted by the organisation’s current rules and procedures. The nurses utilise communication techniques to sustain staff relationships and wellbeing as well as patient standards. This implies that the leadership strategies are directed at advancing the vision and ensuring that it is understood, and that its implementation in the short-term has benefited from a lot of help from extra resources.

*NLs #04, 11, 14, 16, 17, 20, 21, 23, 27, 28, 31, and 32: Supported the values of having corporate communication by “using the English only policy”.*

*NL #07: “Talking to staff nurses in a manner that they will not misunderstand and feel bad by preventing to talk about them in my own language”.*

*NL #15: “Sometimes miscommunication related to language and culture arise but at the end policies of the hospital emerge and make us all equal”. “I use to communicate with different national adding some cultural sensitivity like using only the approved mode of communication in this organization”.*

*SNs #7, 16, and 26: “Showing deep understanding in speaking with Arabs and non-Arab where the nurse leader addresses the team only in English language so that everyone can understand”.*

*SN #8: “Our nurse leader stresses the importance of communication due to the presence of different nationalities with various languages where she reinforces to all of us the use of English language specially during endorsement and even normal chatting our colleagues”.*

*SN #11: “My leader has a great communication skill in relaying a message to all staff and He always use the English policy only as he knows we are multicultural, However, sometimes he uses Arabic to emphasize a phrases/ sentence to Arabic speakers to help them fully understand the topic being discussed”.*

*SN #14: “Value of a nurse leader should be excellent in their communication skills relaying all critical information in approved avenue of communication such as staff meeting, huddle, email or social network groups”.*

### 3.4. Quantitative Phase: Effective Values of Nurse Leaders Perceived as Vital by Nurse Leaders and Staff Nurses 

The survey conducted among the 571 nurses showed that the congruence in perceived values of nurse leaders, as categorised into six themes and their subthemes, was further affirmed by the mean scores ranging from 3.26 to 4.00 for each theme and subtheme, indicating that they are vital and cannot be absent (Table 4). 

Significant at *p*-values equal to or less than 0.05.

#### Significant Differences in the Perceived Nurse Leader Values 

Further statistical analysis done showed that there were no statistically significant differences between staff nurses and nurse leaders in terms of their perceptions of multicultural-related factors, as demonstrated by results of the one-way ANOVA test (Table 5). This indicates that regardless of whether the nurses they are staff nurses or nursing leaders, their perceptions of multicultural-related factors do not change.

## 4. Discussion

This study explored the values that a nurse leader must have to effectively achieve the goals of the institution and ensure safe and high-quality patient care, as perceived by both the nurse leaders themselves and the staff nurses under their leadership. Furthermore, an objective fit of the congruence of the perceived values was performed. 

Cascading deference consists of the subthemes of self-respect, intercultural courtesy, and corporate esteem, which not only emerged as being vital to both staff nurses and nurse leaders, but was also among the top values sought in leaders for work settings where the employees comprise several nationalities with varied cultures and languages. Deference is defined by the Cambridge dictionary [18] as “respect shown for another person especially because of that person’s experience, knowledge, age, or power”. This is similar to the context of deference sought by the nurses in this study, where they considered deference as a distinct moral duty of every individual to have positive attitudes toward human beings’ worth, concerns, and dignity, regardless of race, culture, or religion. In a culturally diverse work setting, the universal language is deference or respect. It is believed that respect and courtesy shown by the leaders toward the staff (and vice versa) as a leadership value and a corporate culture ensures a safe and comfortable work milieu where the staff from different countries can feel secure and inspired to deliver the services expected from them. The respect that they have for themselves drives nursing leaders to excel in the outstanding field of nursing [19]. Moreover, this demonstrates the presence of effective communication among intercultural teams and the provision of high-quality, secure patient care [20]. Nurses need to be more aware of both their own cultural perspectives and the cultural factors that affect intercultural civility [21]. Leng et al. (2021) found that nurses with high self-esteem have more resources for career flexibility and are more content with their jobs [22].

The subthemes trust, integrity, honesty and transparency, and fairness comprise the theme paragon of probity, which emerged as vital for nurse leaders, as mentioned by both staff nurses and nurse leaders. Both nurse leaders and staff nurses expect their leaders to be epitomes of honesty and integrity. Wong et al. (2010) found that a leader’s dependable acts can promote an environment where nurses are driven to accomplish their duties [23]. Integrity is favourably connected with trust in the nursing leaders and the senior management team, according to Mayer et al. (2012) [24]. Honesty and openness refer to the ability of nurse leaders to foster an environment in which each team member feels obligated to uphold the objective of keeping patients safe. Yashdanshenaz et al. (2022) found a positive influence of integrity and ethical values of leaders on the success of the employees, as well as on their psychological gains; moreover, they found an indirect effect of the staff’s perceptions about ethical leadership [25].

Professional competence, with the subthemes professionalism, mentorship, expertise, and accountability, was also perceived by both staff nurses and nurse leaders as being among the most vital leadership qualities. Leaders play a significant role in the achievement of the goals of the institution while also ensuring the welfare of the employees. Their role is crucial in the success of the organisation; hence, their professional competence is imperative. Competence is defined by the US National Library of Medicine as “The capability to perform the duties of one’s profession generally, or to perform a particular professional task, with skill of an acceptable quality” [26]. Leaders earn the trust and respect of both superiors and subordinates even before becoming leaders, through their track record and by showing competence in the performance of their duties. The expertise and professionalism shown in their examples inspire their subordinates to grow professionally. The professional competence of leaders in areas such as mentoring, among others, fosters a safe work milieu, strengthens the faith and sense of security among the employees, enhances productivity, cultivates mindfulness, and improve the employees’ job satisfaction and their trust toward their leader and company [27,28]. As mentors, the leaders focus not only on the technical or procedural skills, but also on soft skills such as motivating subordinates by acknowledging their efforts and contributions, while providing individual feedback [29]. This leadership style is seen as an effective organisational tool to motivate and promote innovative behaviours in workers.

The nurses think that it is important to provide patients with high-quality care while adhering to the values of accountability, respect, and integrity. This suggests that nurse leaders provide the best setting for professional growth in clinical practice. According to Bang and colleagues (2011), the foundation of professional development is the achievement of professional values [30]. The decision to stay in the nursing profession is influenced by excellent mentor experiences, and staff nurses’ experiences of practice learning are greatly influenced by the mentor’s support role [31]. For nurse leaders and managers, the environment in which the nurses work may offer a flexible way to cultivate nursing expertise and attract and retain nursing specialists [32]. The nurse leader is expected to be accountable by taking full responsibility not only for her own actions, but also for the choices, actions, and omissions of their subordinates as they relate to ongoing learning, maintaining expertise, and preserving high standards of both patient care and professional values [33]. Nursing accountability also entails being held accountable to those who are impacted by one’s nursing practice [34]. Finally, understanding the diversity of culture, behaviour, practices, and even professional background and health practices of staff from several different countries is crucial in safe practice.

The theme of compassionate presence, with the subthemes solicitous, humility and gratitude, steadfast zeal, and emotional balance, emerged as vital. To be compassionate, one must be attentive of and empathise with the suffering of others, as well as being aware of their feelings and understanding how they affect them [35]. The nursing leaders can create an environment where the staff’s dispositional humility evolves and their own character blooms in terms of humility and appreciation. This suggests that the nurses can convey their gratitude to others, are willing to learn from their mistakes, and do not overly flaunt their rank or accomplishments. To improve connections with others, understanding of one’s own limits, appreciation and acceptance of others and their goals, being charitable, and the embodiment of the virtue of humility throughout one’s life are presumed to be essential [36]. Happiness in assisting others can be experienced by nurses as they pursue their professional and personal development (steadfast zeal). It can be inferred that nurses have a strong preference for certain activities that they find enjoyable and worthy of their time and effort. The connection between the concept of passion and work activity is underlined in the study of Marsh et al. (2013), underscoring the vital role that this theory plays in industrial and organisational psychology [37]. As a result, Lavigne et al. (2012) asserted that the dualistic model of passion can be used to analyse a person’s involvement in both their work and other activities, and they emphasised the need to consider both dimensions. Furthermore, nurse leaders’ emotional balance is reflected in how they can manage both their own and other people’s emotions [38]. This suggests that nurses make wiser decisions, treat their patients better, form closer relationships with patients and their families, and improve their interpersonal skills when they are able to regulate their emotions.

Team diversity and inclusion has only one subtheme—collaboration and cooperation—which turned out to be vital for both staff nurses and nurse leaders. In a diverse community, this value is almost expected to emerge. Ensuring that the work is carried out in a group with multiple languages and cultures, without the peculiarities, is a major challenge. Hence, a leader who builds teamwork is considered to be wise. Collaboration and teamwork among healthcare professionals have been shown to be crucial for the delivery of high-quality healthcare coverage, as well as for improved patient and professional contentment [39]. Healthcare professionals gain from inter-professional teamwork, and organisations can more affordably serve patients with services that are more extensive and efficient [40]. Leaders ensure that their staff are provided with training for professional development, which is required for successful teamwork and collaboration in the healthcare sector [41]. Nurses look for leaders who prioritise teamwork in the institution.

Calibrated communication includes two subthemes: communication between nurse leaders and staff nurses, and corporate communication, which were considered by both leaders and staff as being vital. Communication skills are indispensable among leaders [42,43]. These results suggest that instructional leadership by nurse leaders may improve performance and aid the hospital in achieving its objectives. According to Andrade Vasconcelos and colleagues (2017), nursing leverages the interpersonal communication that occurs when providing care for patients as a tool for knowing and behaving [44]. The nursing team’s ability to communicate is directly hindered in their work [45]. Leaders who rarely communicate with their staff, or who provide poor feedback or information and hardly listen to their subordinates, can certainly demoralise and demotivate their staff [46]. Good communication between leaders and employees is positively associated with organisational success [47,48]. Studies have shown a significant effect of communication with the employees. Furthermore, the quality of the communication process reflects the kind of leadership, which ultimately affects the organisational performance [49,50].

Corporate communication was considered by both leaders and staff as being essential. The leaders have to give attention to systematic or strategic communication, which refers to the calculated use of communication by the leaders to fulfil the organisational goals [50,51]. Chitrao (2014) found a positive association between leaders’ good communication and decreases in the rate of staff grievances, as well as increases in productivity, profits, and job satisfaction [51]. Leaders and subordinates need leaders and managers who are competent in strategic and systematic communication to effectively achieve their institutional goals [52,53,54].

## 5. Conclusions

It can be concluded that despite the diverse nursing workforce in this tertiary hospital, congruence exists in the perceived values of effective leaders. Both nurse leaders and staff nurses, despite their varied nationalities, cultures, languages, and values, shared similar perceived effective values that they expect from the nurse leaders. The existence of congruent nurse leadership values determined in this study indicates that these values have universal worth and can be a potent means of a remarkable coalescence of forces from a diverse workforce to achieve institutional goals.

It is recommended that the nurse leaders from a multicultural mix of nursing staff should be cognisant not only of the diversity of culture and language, but also of the values held by their staff. This will ensure mutual understanding and motivation to deliver safe and high-quality services.

The findings of this study can be primarily valuable to the nurse leaders, who have the responsibility of ensuring interracial agility among their staff by investing in the congruent values of nurse leaders and staff. Ultimately, the patient will benefit from the provision effective and efficient healthcare services by a unified and integrated nursing staff.

Further studies could be carried out in the same institution on the integration of the congruent nurse leader values explored in this study into the nurse leaders’ orientation or development programs, which could be further evaluated over time against patient safety and the nurses’ job engagement as outcomes. Similar studies could explore the objective and subjective fitting of values among nurse leaders and their staff, as there is a dearth of literature in this area.

## 6. Limitations of the Study

This study was conducted during the COVID-19 pandemic, when social distancing and other restrictions were in place. Hence, the interviews were mostly conducted online, where body language and some emotions that could provide more meaning to the verbalisations were difficult to capture.

## Figures and Tables

**Table 1:** Sequential mixed-methods design of the study.

Phase	Participants	Instrument	Analysis	Outcomes
Qualitative data collection	70	Individual in-depth interviews with nurse leaders and staff nurses	Thematic analysis by Colaizzi	Congruent values (themes) of effective nursing leadership
Quantitative phase	571	Survey questionnaire on leadership values developed from the themes in phase 1	Mean scoresANOVA	Values of effective leaders in a multicultural medical city

**Table 2:** Sociodemographic profile: qualitative phase.

Country	Nurse Leader Participants	Staff Nurse Participants
Philippines	11	20
India	8	5
Saudi	6	9
Jordan	4	2
South Africa	4	1
Total	33	37
Gender	Nurse leader participants	Staff nurse participants
Male	4	8
Female	29	29
Total	33	37
Hospitals	Nurse leader participants	Staff nurse participants
Outpatient department	3	2
Rehabilitation hospital	3	6
Main hospital	5	5
Comprehensive cancer centre	2	4
Women’s specialised centre	8	8
National Neuroscience Institute	2	1
Critical care nursing administration	2	1
King Salman Heart Centre	4	3
Children’s specialised hospital	2	3
Total	33	37

**Table 3:** Sociodemographic profile: quantitative phase (*n* = 571).

	Frequency	Percent
**Hospitals and Centres**		
Rehabilitation hospital	132	23.1
Women’s specialised hospital	99	17.3
Main hospital	76	13.3
National Neuroscience Institute	52	9.1
Outpatient department	43	7.53
Critical care	36	6.3
Emergency department	36	6.3
King Salman Heart Centre	34	5.95
Children’s specialised hospital	19	3.3
Comprehensive cancer centre	17	3
Others	12	2.62
Operating theatres	15	2.1
**Total**	**571**	**100**
**Age**		
23–30 Years old	126	22.1
31–38 Years old	266	46.6
39–46 Years old	101	17.7
47–54 Years old	75	13.1
55 Years old and above	3	0.5
Total	571	100
**Nationality**		
Greater Middle East countries		
Saudi Arabia	23	66%
Jordan	7	20%
Syria	1	3%
Egypt	1	3%
Pakistan	3	9%
Asian countries		
Philippines	411	78%
Malaysia	4	1%
South Korea	1	0%
India	111	21%
Western countries		
United States	1	11%
Kosovo	1	11%
South Africa	7	78%
**Country groupings**		
Greater Middle East countries	35	6.13%
Asian countries	527	92.29%
Western countries	9	1.58%
Total	571	100%

**Table 4:** Weighted mean distribution of effective values of nurse leaders perceived as vital by nurse leaders and staff nurses (*n* = 571).

	*Nurse Leader*		*Staff Nurse*	
	Mean	Verbal Interpretation	Mean	Verbal Interpretation
**Theme No. 1 Cascading Deference**				
Self Respect	3.83	Vital and Cannot be Absent	3.84	Vital and Cannot be Absent
Intercultural Courtesy	3.72	Vital and Cannot be Absent	3.73	Vital and Cannot be Absent
Corporate Esteem	3.86	Vital and Cannot be Absent	3.85	Vital and Cannot be Absent
***Weighted Mean***	***3.8***	Vital and Cannot be Absent	***3.81***	***Vital and Cannot be Absent***
**Theme No. 2 Paragon of Probity**				
Trust	3.88	Vital and Cannot be Absent	3.85	Vital and Cannot be Absent
Integrity	3.78	Vital and Cannot be Absent	3.81	Vital and Cannot be Absent
Honest/Transparency	3.86	Vital and Cannot be Absent	3.86	Vital and Cannot be Absent
Fairness	3.83	Vital and Cannot be Absent	3.86	Vital and Cannot be Absent
***Weighted Mean***	***3.84***	***Vital and Cannot be Absent***	***3.85***	***Vital and Cannot be Absent***
**Theme No. 3 Professional Competence**				
Professionalism	3.85	Vital and Cannot be Absent	3.84	Vital and Cannot be Absent
Mentorship	3.83	Vital and Cannot be Absent	3.86	Vital and Cannot be Absent
Expertise	3.86	Vital and Cannot be Absent	3.86	Vital and Cannot be Absent
Accountability	3.83	Vital and Cannot be Absent	3.85	Vital and Cannot be Absent
***Weighted Mean***	***3.84***	***Vital and Cannot be Absent***	***3.85***	***Vital and Cannot be Absent***
**Theme No. 4 Compassionate Presence**				
Solicitous	3.78	Vital and Cannot be Absent	3.83	Vital and Cannot be Absent
Humility and Gratitude	3.75	Vital and Cannot be Absent	3.79	Vital and Cannot be Absent
Steadfast Zeal	3.81	Vital and Cannot be Absent	3.84	Vital and Cannot be Absent
Emotional Balance	3.81	Vital and Cannot be Absent	3.84	Vital and Cannot be Absent
***Weighted Mean***	***3.79***	***Vital and Cannot be Absent***	***3.83***	***Vital and Cannot be Absent***
**Theme No. 5 Team Diversity And Inclusion**				
Collaboration and Cooperation	3.78	Vital and Cannot be Absent	3.82	Vital and Cannot be Absent
**Theme No. 6 Calibrated Communication**				
Communication between Nurse Leader and Staff Nurse	3.83	Vital and Cannot be Absent	3.81	Vital and Cannot be Absent
Corporate Communication	3.8	Vital and Cannot be Absent	3.84	Vital and Cannot be Absent
***Weighted Mean***	***3.82***	***Vital and Cannot be Absent***	***3.83***	***Vital and Cannot be Absent***

**Table 5:** Test of significant differences between the nurse leaders’ and staff nurses’ perceived values of nurse leaders (*n* = 571).

		95% Confidence Interval for Mean													
		N	Mean	Std. Deviation	Std. Error	Lower Bound	Upper Bound	Min	Max		Sum of Squares	df	Mean Square	F	Sig.
	Staff Nurse	466	57.17	4.647	0.215	56.75	57.6	21	60	Between Groups	4.571	1	4.571	0.211	0.646
**Cascading Deference**	Nurse Leaders	105	56.94	4.696	0.458	56.03	57.85	39	60	Within Groups	12334.6	569	21.678		
	Total	571	57.13	4.653	0.195	56.75	57.51	21	60	Total	12339.1	570			
	Staff Nurse	466	46.18	3.651	0.169	45.84	46.51	12	48	Between Groups	0.853	1	0.853	0.069	0.794
**Paragon of Probity**	Nurse Leaders	105	46.08	2.918	0.285	45.51	46.64	37	48	Within Groups	7084.96	569	12.452		
	Total	571	46.16	3.526	0.148	45.87	46.45	12	48	Total	7085.81	570			
	Staff Nurse	466	46.24	3.632	0.168	45.91	46.57	12	48	Between Groups	1.316	1	1.316	0.103	0.748
**Professional Competence**	Nurse Leaders	105	46.11	3.268	0.319	45.48	46.75	33	48	Within Groups	7245.19	569	12.733		
	Total	571	46.22	3.566	0.149	45.92	46.51	12	48	Total	7246.5	570			
	Staff Nurse	466	53.5	4.41	0.204	53.09	53.9	31	56	Between Groups	26.191	1	26.191	1.304	0.254
**Compassionate Presence**	Nurse Leaders	105	52.94	4.785	0.467	52.02	53.87	37	56	Within Groups	11424.1	569	20.078		
	Total	571	53.39	4.482	0.188	53.03	53.76	31	56	Total	11450.3	570			
	Staff Nurse	466	11.45	1.051	0.049	11.35	11.54	5	12	Between Groups	0.792	1	0.792	0.709	0.4
**Team Diversity and Inclusion**	Nurse Leaders	105	11.35	1.083	0.106	11.14	11.56	6	12	Within Groups	635.226	569	1.116		
	Total	571	11.43	1.056	0.044	11.34	11.52	5	12	Total	636.018	570			
	Staff Nurse	466	22.97	1.909	0.088	22.79	23.14	12	24	Between Groups	0.548	1	0.548	0.152	0.696
**Calibrated Communication**	Nurse Leaders	105	22.89	1.831	0.179	22.53	23.24	17	24	Within Groups	2044.08	569	3.592		
	Total	571	22.95	1.894	0.079	22.8	23.11	12	24	Total	2044.63	570			

## Data Availability

The data that support the findings of this study are available from the corresponding author upon reasonable request.
